# Is medical home care adequacy associated with educational service use in children and youth with autism spectrum disorder (ASD)?

**DOI:** 10.1186/s12887-022-03776-3

**Published:** 2023-01-09

**Authors:** Sabrin Rizk, Emmanuel Ngui, Teal W. Benevides, Victoria A. Moerchen, Mary Khetani, Kris Barnekow

**Affiliations:** 1 Department of Occupational Therapy, College of Applied Health Sciences, University of Illinois Chicago, 1919 West Taylor Street, AHSB 744, Chicago, IL 60612 USA; 2 Community & Behavioral Health Promotion, University of Wisconsin-Milwaukee, Joseph J. Zilber School of Public Health, 1240 North 10th Street, Rm 417, Milwaukee, WI 53205 USA; 3 Department of Occupational Therapy, College of Allied Health Sciences, Augusta University, 987 St. Sebastian Way, EC-2324, Augusta, GE 30912 USA; 4 Department of Rehabilitation Sciences & Technology, College of Health Sciences, University of Wisconsin-Milwaukee, 3409 North Downer, Pavilion, PT Suite, Rm 366, PO Box 413, Milwaukee, WI 53201 USA; 5 Department of Occupational Therapy, College of Applied Health Sciences, University of Illinois Chicago, 1919 West Taylor Street, 316A, Chicago, IL 60612 USA; 6 Department of Rehabilitation Sciences & Technology, College of Health Sciences, University of Wisconsin-Milwaukee, 2400 East Hartford Avenue, Enderis Hall, 979, PO Box 413, Milwaukee, WI 53201 USA

**Keywords:** Autism spectrum disorders, Education services, Health services, Pre-school children, School-age children, Adolescents

## Abstract

**Background:**

The American Academy of Pediatrics (AAP) recommends medical home care for children and youth with autism spectrum disorder (ASD) for health needs. Children and youth with ASD also receive educational services for cognitive, social, and behavioral needs. We measured whether inadequate medical home care was significantly associated with current educational service use, controlling for sociodemographic factors.

**Methods:**

We analyzed the 2016/2017 National Survey of Children’s Health (NSCH) on 1,248 children and youth with ASD ages 1–17. Inadequate medical home care was operationalized as negative or missing responses to at least one medical home component. Educational service use was defined as current service use under individualized family service plans (IFSP) and individualized education programs (IEP).

**Results:**

Inadequate medical home care was significantly associated with higher likelihood of current educational service use (*aOR* = 1.95, 95% CI [1.10, 3.44], *p* = 0.03). After adjustment, older children (*aOR* = 0.91, 95% CI [0.84, 0.99], *p* = 0.03), lower maternal health (*aOR* = 0.52, 95% CI [0.29, 0.94], *p* = 0.03), and children without other special health care factors (*aOR* = 0.38, 95% CI [0.17–0.85], *p* = 0.02) had significantly lower odds of current educational service use.

**Conclusions:**

Inadequate medical home care yielded higher odds of current educational service use. Child’s age, maternal health, and lack of other special health care factors were associated with lower odds of current educational service use. Future research should examine medical home care defined in the NSCH and improving educational service use via medical home care.

**Supplementary Information:**

The online version contains supplementary material available at 10.1186/s12887-022-03776-3.

## Background

Children and youth with autism spectrum disorder (ASD) have complex primary and specialty medical care needs and additionally may use other community and supportive services. Families of children and youth with ASD navigate multiple, intricate service systems, from early childhood through young adulthood. The American Academy of Pediatrics (AAP) asserts the medical home as a best practice standard of care for all children, and particularly children and youth with ASD, for accessible, continuous, comprehensive, family centered, coordinated, compassionate, and culturally effective care [2].

Prior health services research findings identified benefits of medical homes for primary and specialty care, which is associated with earlier ASD detection and reduced wait times for establishing ASD diagnoses once detected [18, 22] annual preventative medical care visits [27] and decreased emergency department (ED) utilization [16]. High-quality medical homes have been associated with greater likelihood of met service needs for children and youth with ASD (e.g., occupational, physical, and speech therapy) and supportive services (e.g., special equipment, transportation, home health, and respite) [8]. Children and youth receiving family centered and coordinated medical home care had fewer unmet service needs [34]. Yet, children and youth with ASD are still less likely to receive medical home care, which is associated with greater unmet service needs, service gaps, costly care inclusive of ED utilization, and increased family responsibilities [9].

In the US, medical home disparities for children and youth with ASD have been attributed to providers’ limited ASD knowledge and training [38] and in the setting of financial, social, linguistic, and cultural barriers of the families of children and youth with ASD [43]. Racial disparities in medical home care access have been shown for children and youth with ASD (i.e., Black, non- Hispanic, Hispanic, or identified as ‘other race’ (i.e., American Indian, Alaska Native, Asian, Native Hawaiian, or other Pacific Islander) compared to White, non-Hispanic children and youth with ASD [11, 27]. Children and youth with ASD with public insurance have also had lower likelihood of receiving medical home care [44].

Understanding the barriers that children and youth with ASD uniquely encounter may manifest in key medical home functions that should collectively promote access (e.g., addressing parental developmental concerns, adherence to recommended ASD screening schedule, referrals for ASD diagnostic assessments, timely identification, and coordinating with early intervention or special education programs) [2]. Although the AAP’s *Bright Futures* guidelines endorses periodic developmental and ASD-specific screening [14] there is mixed evidence on age of ASD identification, [20] underscoring challenges with early identification of ASD and lost early intervention referrals through the medical home pathway for those children identified after 3 years. Additionally, children may receive ASD-specific screenings on schedule, but experience delays in ASD diagnostic assessments and early intervention eligibility determination [25]. Families of children and youth with ASD who express challenges in sharing their developmental concerns may delay referrals for ASD diagnostic assessments and early intervention eligibility determination [30]. Sociodemographic factors (e.g., lower educational attainment, unemployment, race/ethnicity, and socioeconomic status (SES)) have been cited as challenges to early intervention access [26].

Children and youth with ASD ages 3 through 21 with qualifying disabilities in public school systems receive services under individualized education programs (IEP) according to US federal law [42]. Medical diagnosis of ASD versus educational ASD classification for special education programming are distinct processes requiring families’ understanding of the differences in the service systems’ requirements. Diagnostic criteria for medical ASD are based on the Diagnostic and Statistical Manual of Mental Disorders (DSM), [3] whereas the US educational ASD classification is based on the Individuals with Disabilities Education Act (IDEA) Part B for special education service eligibility. In addition, skilled therapies outlined in IEPs (e.g., occupational, speech and physical therapy) are educationally based “related services” [42] that are different from medically based therapies, although this distinction can confuse medical home care providers and families [17].

Medical home care providers play an important role in supporting access to educational services for children and youth with ASD and their families, such as by initiating referral into early intervention or school-based services to determine service eligibility [28]. As such, medical homes that are better integrated with educational services are important in promoting children’s overall developmental and cognitive well-being. Children and youth’s health statuses and educational outcomes contribute to children's overall health and well-being, and different providers are integral for addressing the educational needs for children and youth with ASD, including medical home care providers, educational personnel, and families/caregivers. While AAP policy and prior conceptual work support the relationship between medical home care and educational service use [1], there is lack of quantifiable population-based evidence on the association between medical home care and educational service use for the pediatric ASD population specifically [39]. Therefore, the purpose of this study is two-fold: 1) to establish the association between medical home care adequacy and the likelihood of current educational service use for children and youth with ASD; and 2) to examine sociodemographic factors that attenuate the association between medical home care adequacy and educational service use. We hypothesized that reported inadequate medical home care will be associated with decreased current educational service use and attenuated by select sociodemographic factors.

### Methods

We analyzed cross-sectional data from the 2016–2017 National Survey of Children’s Health (NSCH) [12] to evaluate the relationship between inadequate medical home care and current educational service use in children and youth with ASD. The NSCH is a nationally representative survey of parents or guardians (caregivers) of children and youth ages 0 to 17 years old in the US and District of Columbia to assess children’s physical and mental health, health care access, family characteristics, neighborhoods, school, and social conditions. The 2016 NSCH was conducted from June 2016 to February 2017, and the 2017 NSCH was conducted from August 2017 to February 2018.

The NSCH uses a complex survey design with a multi-level stratified sampling, primary sampling units (PSU), and survey weights to adjust for the sampling approach [41]. Survey sampling weights were adjusted to control for nonresponse bias in the individual 2016 and 2017 NSCH [41]. Caregivers completed the NSCH screening questionnaire to determine if children resided in their households, and proceeded to identify children and youth with special health care needs (CSHCN) [33] through a validated CSHCN screener [10], by responding affirmatively to at least one of five criteria: 1) need or use of prescription medications, 2) elevated service needs, 3) functional limitations, 4) therapeutic service use, and 5) emotional, developmental, or behavioral problems warranting treatment or counseling. The CSHCN screener further assessed if affirmative response(s) were attributed to a medical, behavioral, or other health condition expected to last ≥ 12 months. Eligible caregivers advanced to age specific NSCH topical questionnaires, yielding data on all current study variables [12].

### Sample

Children and youth ages 1–17 whose caregivers reported positive or valid responses to, “Has a doctor or other health care provider EVER told you that this child has ASD or autism spectrum disorder (ASD)?” were in the analytic sample (*n* = 1,248).

### Dependent variable

The dependent variable was current educational service use under an IFSP or an IEP. The 2016/2017 NSCH asked, “Has this child EVER had a special education or early intervention plan?” followed by, “Is this child CURRENTLY receiving services under one of these plans?” Current educational service use was defined as receiving services currently under one of these plans.

### Independent variable

**Fig. 1:**
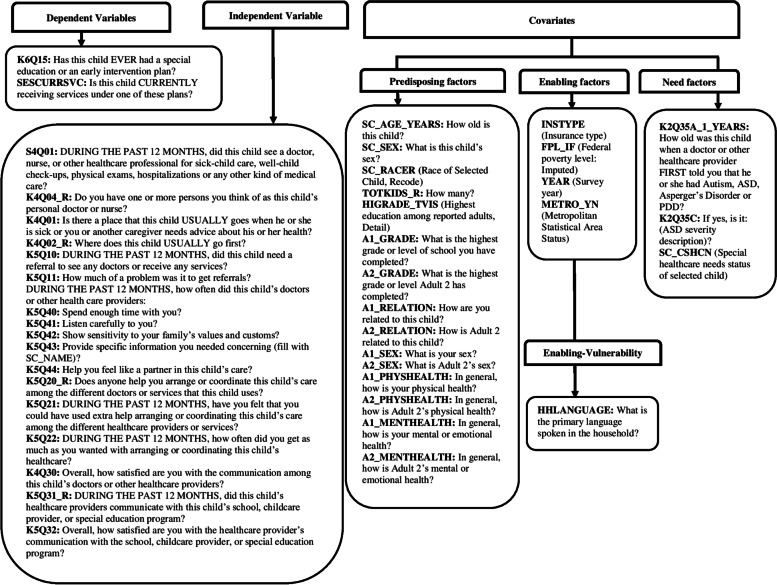
The 2016/2017 NSCH medical home care survey questions are grouped by the five medical home care components. Variable coding are italicized and in parentheses

The independent variable was inadequate medical home care to reflect medical home care for children and youth with ASD [27]. As shown in Fig. 1, the 2016/2017 NSCH used a composite measure of medical home care based on a series of questions across five components: 1) personal doctor or nurse, 2) usual source of sick care, 3) family centered care, 4) referral problems when needed, and 5) effective care coordination when needed.

The NSCH medical home care question route began by affirming whether caregivers had individuals they considered the child’s personal doctor or nurse. Usual source of sick care was operationalized as having a usual place to go when the child is sick, except the ED. Family centered care used five intermediate variables to describe providers’ interactions: 1) spending enough time with the child, 2) listening carefully, 3) sensitivity to family values, 4) providing specific information, 5) and helping families feel like a partner in the child’s care. Children and youth with ASD received family centered care if their caregivers reported “Always” or “Usually” for each family centered care intermediate variable. Referral problems when needed was defined as caregivers reporting needed referrals and not experiencing problems getting referrals. Having effective care coordination, was defined as caregivers’ reported need for assistance with care coordination, “Usually” receiving assistance, and were “Very Satisfied” with communication between the child’s doctor, other health care providers, schools, child care, or special education program.

We employed a stringent criterion to operationalize adequate medical home care as positive and valid caregiver responses across the five medical home components to understand how this rigorous definition is postured on the issue of coordinated health care and educational services for children and youth with ASD. Since adequacy of medical home care is relative to service need, caregivers who reported children “Did not have health care visit in the past 12 months,” “Did not need referrals during the past 12 months,” or “Did not need coordinated care or had less than 2 services during the past 12 months” were also coded as having adequate medical home care. Inadequate medical home care was operationalized as negative or missing responses to one or more of the five medical home components to methodologically decouple missingness (i.e., missing to all versus missing some or one) and preserve a stringent medical home composite measure. We used a conservative measure of “Missing” if children and youth with ASD had invalid responses to all five medical home components and excluded from analyses.

### Conceptual framework and covariates

The Behavioral Model for Vulnerable Populations, an expansion of the Andersen Behavioral Model of Health Services Use, with four domains: predisposing, enabling (including enabling-vulnerability), and need [4, 5, 7, 19] guided this study. The original Andersen model has been commonly used to examine factors affecting health service use [6], and a smaller body of work has also demonstrated its utility with examining factors affecting educational service use [23]. Covariates were identified based on prior research and entered sequentially in the multivariate models based on the four Andersen Behavioral Model of Health Services Use domains.

Sociodemographic information was based on caregiver report for predisposing, enabling (including enabling-vulnerability), and need factors. Predisposing factors expected to affect the likelihood of educational service use included child sex, age (continuous), total number of children in household (1–2; ≥ 3), child race/ethnicity (White, Non-Hispanic; Black, Non-Hispanic; Other), adult education level (< high school; > high school), maternal health status (excellent/very good; < excellent/very good), and family structure (two parents (married/unmarried); single parent, other, none reported).

Enabling factors expected to facilitate current educational service were insurance type (private, public, or unspecified insurance) and family federal poverty level (FPL) (≤ 199%;> 199%) [11, 13, 35, 44]. Vulnerability was assessed using the primary language spoken at home categorized as English, Spanish, and other languages.

Need factors expected to increase the propensity for current educational service use included child age at ASD diagnosis (continuous), ASD severity (mild/moderate; severe), and CSHCN factors (ASD, CSHCN; ASD, non-CSHCN).

### Analyses

We used Stata 15.1 to apply survey weights appropriate for the subpopulation, the multi- stage sampling design, and correct variance estimation [40]. Predictor variables were checked for multicollinearity and no assumptions were violated. Bivariate comparison of all dichotomous sociodemographic covariates and current educational service use was performed with chi-square tests. We retained statistically significant covariates (*p* < 0.05), and all non-significant covariates (*p* > 0.05) with demonstrated theoretical or clinical significance [21] (i.e., child race/ethnicity, adult education level, family structure, insurance type, and family FPL) from previous health or educational service literature [23].

Predisposing, enabling (including enabling-vulnerability), and need factors were sequentially added during multivariate analyses. We began with a model to predict current educational service use from inadequate medical home care (Model 1), followed by adding the predisposing factors (Model 2). Model 3 added enabling factors, Model 4 tested the enabling-vulnerability factor, and the full model included the need factors. We obtained adjusted odds ratios (*aOR*), significance levels (*p* < 0.05), and the 95% confidence interval (CI) to assess statistically significant predictors in multivariate models.

This study was exempt by the University of Wisconsin-Milwaukee’s Institutional Review Board.

## Results

### Sample characteristics

As shown in Table 1, the demographic characteristics included 1,248 children and youth with ASD. Most of the sample with inadequate medical home care were non-Hispanic White (53.5%), publicly insured (60.5%), in the ≤ 199% FPL (55.2%), had mild/moderate ASD severity (89.6%), and had other caregiver reported CSHCN factors (95.6%). More than half of those with inadequate medical home care were males, had 1–2 children in their household, lower maternal health, and from single parent or other family structures. Children and youth with ASD with adequate and inadequate medical home care significantly differed by race/ethnicity, insurance type, poverty level, ASD severity, and CSHCN factors.

**Table 1:** Characteristics of children and youth with ASD by adequacy of medical home care

	**Adequate**	**Inadequate**	***p* value**
	(*n* = 325), % [95% CI]	(*n* = 923), % [95% CI]	
**Predisposing factors**			
Child’s sex			
Male	80.61 [70.94–87.62]	77.07 [67.67–84.37]	.558
Female	19.39 [12.38–29.06]	22.93 [15.6–32.3]	
Child’s age, mean y	11.00 [10.2–11.85]	10.24 [9.63–10.84]	
Total children in household			
1–2	67.23 [56.94–76.09]	71.39 [63.73–77.99]	.494
≥ 3	32.77 [23.91–43.06]	28.61 [22.01–36.27]	
Child’s race/ethnicity			
White, Non-Hispanic	75.15 [66.24–82.34]	53.49 [44.2–62.5]	.001^b^
Black, Non-Hispanic	11.67 [6.50–20.07]	16.55 [11.76–22.78]	
Other	13.18 [8.51–19.86]	29.96 [20.33–41.76]	
Overall maternal health status			
Excellent or very good	51.63 [42.08–61.05]	49.88 [41.13–58.64]	.793
< Excellent or very good	48.37 [38.9–57.92]	50.12 [41.36–58.87]	
Adult Education Level			
> High School	80.68 [70.60–87.89]	68.44 [58.84–76.68]	.060
≤ High School	19.32 [12.11–29.40]	31.56 [23.32–41.16]	
Family structure			
Two parents, married/unmarried	82.46 [73.66–88.76]	77.29 [70.73–82.74]	.307
Single parent, other, none reported	17.54 [11.24–26.34]	22.71 [17.26–29.27]	
**Enabling factor** Insurance type			
Private	62.25 [52.58–71.03]	37.00 [30.05–44.54]	.001^b^
Public	34.34 [26.14–43.58]	60.46 [52.67–67.75]	
Unspecified	3.42 [0.65–15.97]	2.54 [1.33–4.81]	
Federal poverty level (FPL)			
> 199% FPL	66.70 [56.87–75.26]	44.82 [36.84–53.08]	< .001^c^
≤ 199% FPL	33.30 [24.74–43.13]	55.18 [46.92–63.16]	
**Enabling-vulnerability factor**			
Primary household language			
English	91.96 [82.39–96.55]	82.68 [71.79–89.96]	.185
Spanish	5.01 [1.45–15.88]	13.90 [7.11–25.39]	
Other	3.03 [1.11–8.01]	3.42 [1.51–7.59]	
**Need factors ** Autism severity			
Severe	5.03 [2.61–9.48]	10.38 [7.30–14.58]	.045
Mild/moderate	94.97 [90.52–97.39]	89.62 [85.42–92.70]	
CSHCN status			
ASD, CSHCN	87.33 [80.17–92.16]	95.60 [92.73–97.37]	.001^b^
ASD, non-CHSCN	12.67 [7.84–19.83]	4.40 [2.63–7.27]	
Age at autism diagnosis, mean y	4.56 [4.08–5.04]	4.42 [4.05–4.79]	

^a^ *p* < .05

^b^ *p* < .01

^c^ *p* < .001

### Medical home care adequacy and factors associated with current educational service use

We hypothesized that reported inadequate medical home care will be associated with decreased current educational service use. Our hypothesis was unsupported. In Table 2, bivariate analyses yielded a significantly higher percentage of children and youth with ASD with inadequate medical home care and current educational service use, compared to those adequate medical home care (82.5% versus 17.4%). A higher percentage of children and youth with ASD with other CSHCN factors were reportedly using educational services currently, compared to children and youth with ASD but no other CSHCN factors (95.3% versus 4.7%) (Table 2).

**Table 2:** Factors associated with current educational service use among children and youth with ASD

	**No current educational services**	**Current educational services**	***p* value**
	(*n* = 188), % [95% CI]	(*n* = 1,060), % [95% CI]	
Adequacy of medical home care Adequate	31.76 [21.21–44.60]	17.47 [13.71–22.01]	.011^a^
Inadequate	68.24 [55.40–78.79]	82.53 [78.0–86.53]	
**Predisposing factors**			
Child sex			
Male	84.34 [74.69–90.77]	76.63 [67.76–83.65]	.190
Female	15.66 [9.23–25.31]	23.37 [15.4–32.24]	
Child age, mean y	11.78 [10.83–12.73]	10.15 [9.56–10.73]	
Total children in household			
1–2	68.09 [55.42–78.55]	71.00 [63.72–77.34]	.670
≥ 3	31.91 [21.45–44.58]	29.00 [22.66–36.28]	
Child race			
White, Non-Hispanic	67.74 [52.81–79.75]	56.01 [46.96–64.68]	.422
Black, Non-Hispanic	13.35 [4.48–33.64]	15.98 [11.71–21.43]	
Other	18.91 [11.49–29.53]	28.01 [18.84–39.47]	
Overall maternal health status			
Excellent or very good	38.60 [27.42–51.12]	52.22 [44.1–60.23]	.070
< Excellent or very good	61.40 [48.88–72.58]	47.78 [39.77–55.90]	
Adult education level			
> High school	65.26 [50.10–77.84]	71.79[62.63–79.44]	.427
≤ High school	34.74 [22.16–49.90]	28.21 [20.6–37.37]	
Family structure			
Two parents, married/unmarried	79.99 [68.92–87.82]	78.01 [71.84–83.14]	
Single parent, other, none reported	20.01 [12.18–31.08]	21.99 [16.86–28.16]	.728
**Enabling factors**			
Insurance type			
Private	46.01 [33.73–58.80]	41.25 [34.13–48.74]	.785
Public	51.94 [39.16–64.48]	55.93 [48.17–63.41]	
Unspecified	2.04 [.34–11.38]	2.82 [1.43–5.51]	
Federal poverty level (FPL)			
> 199% FPL	55.04 [41.65–67.74]	48.08 [40.11–56.15]	.383
≤ 199% FPL	44.96 [32.26–58.35]	51.92 [43.85–59.89]	
**Enabling-vulnerability factor**			
Primary household language			
English	90.24 [79.10–95.76]	83.51 [73.12–90.41]	.349
Spanish	8.73 [3.48–20.22]	12.75 [6.38–23.86]	
Other	1.03 [.24–4.28]	3.74 [1.81–7.59]	
**Need factors**			
Autism severity			
Severe	7.95 [4.03–15.09]	9.58 [6.73–13.45]	.623
Mild/moderate CSHCN status	92.05 [84.91–95.97]	90.42 [86.55–93.21]	
Autism, CSHCN	86.57 [78.19–92.05]	95.26 [92.41–97.07]	.002^b^
Autism, non-CHSCN	13.43 [7.95–21.81]	4.74 [2.93–7.59]	
Age at autism diagnosis, mean y	5.24 [4.59–5.90]	4.31 [3.97–4.65]	

^a^ *p* < .05

^b^ *p* < .01

^c^ *p* < .001

### Predicting current educational service use from inadequate medical home care

We hypothesized that the association between reported inadequate medical home care and current educational service use would be attenuated by select sociodemographic factors. Our hypothesis was partially supported. Multivariate analyses showed that children and youth with ASD with inadequate medical home care had significantly higher odds of current educational service use (see Appendix, Model 1). After adjusting for predisposing factors, the odds of current educational service use slightly decreased, but remained statistically significant. Older children and youth with ASD and mothers with reported lower overall health (i.e., < less than excellent or very good) had significantly lower odds of current educational service use (see Appendix, Model 2). The enabling factors (including enabling-vulnerability) did not affect the interpretation of inadequate medical home care or the other predisposing factors (see Appendix, Models 3 and 4).

**Fig. 2:**
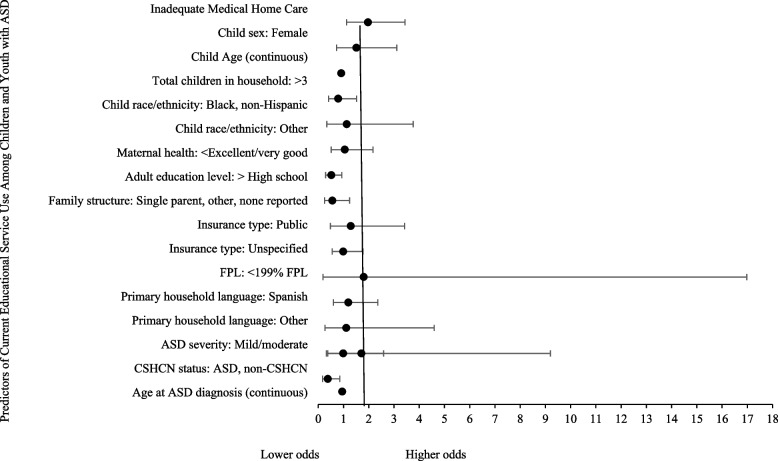
Multivariate logistic regression model predicting current educational service use from inadequate medical home care and controlling for all covariates (*n* = 1, 248*)*

The effect of inadequate medical home care was slightly attenuated in the fully adjusted model (see Fig. 2) but remained statistically significant. Children and youth with ASD receiving inadequate medical home care had significantly higher odds of current educational service use, controlling for selected covariates. Predisposing factors (i.e., child age and maternal health status) were significantly associated with lower odds of current educational service use. Older children and youth with ASD and those with mothers reporting lower overall health were significantly less likely to currently use educational services. Lastly, the presence of other CSHCN factors was associated with significantly lower odds of current educational service use.

## Discussion

To our knowledge, this is the first population-based study establishing the association between medical home care and current educational service use in children and youth with ASD. To overcome the fragmented care between medical home care providers and educational service professionals (e.g., early intervention and special education programming), we departed from the traditional approaches that distinctly attends to one system separately from the other. Our study begins to establish connections between the medical home care and educational service sectors [39]. Their disconnect will perpetuate costly, redundant, and poorer quality and access to coordinated care for children and youth with ASD and their families. Intervening at both systems’ level can and should synergistically feedback and affect processes and outcomes between these services systems. Our hypothesis that inadequate medical home care was associated with decreased current educational service use was not supported. Instead, findings suggested that inadequate medical home care was significantly associated with higher odds of current educational service use, controlling for predisposing, enabling (including enabling-vulnerability), and need factors.

One possible explanation for this unexpected finding is that the medical and educational systems have distinct processes intrinsic to how they are organized and delivered (e.g., referrals, evaluation, eligibility determination, and service delivery). These system differences may contribute to fragmented care and further compound the difficulty families experience as informal care coordinators between intricate service structures. Existing literature has endorsed coordinated care between the medical home and educational services, including early intervention and special education programs [32]. However, the implementation of practice recommendations is challenged by barriers, including additional time needed to coordinate with educational service providers, reimbursement for indirect service delivery, and medical home care provider knowledge of educational services [18, 24]. Additional barriers to medical home coordinated care have included medical home providers’ knowledge on ASD and educational services and attitudes about collaborating with providers external to the primary care environment [38].

Alternatively, this unexpected finding may reflect measurement considerations. For example, the NSCH restricts measurement of current educational service use to current IFSP or IEP receipt; therefore, receipt of additional educational service use cannot be discerned from the survey. Children and youth with ASD may receive additional educational services under Section 504 of the Rehabilitation Act of 1973 [42], such as older children or those with milder condition severity. Since the NSCH medical home care variable is a multidimensional concept with numerous variables yielding a composite measure, future studies can and should examine medical home care components in relationship to the odds of current educational service use [31]. Given their established significance in this study, these subsequent analyses could stratify by child age (e.g., 0–5 versus 6–17 years) and/or CSHCN factors to better elucidate how CSHCN factors bolster the likelihood of educational service use.

Study results strengthen evidence on the role of maternal health status in relationship to medical home care and current educational service use among children and youth with ASD. Patient-centered medical home care has been significantly associated with increased maternal mental health and less parenting stress; however, mothers of children and youth with ASD were less likely to report receiving effective care coordination, thereby increasing maternal stress and decreasing mental health perceptions [29]. Similarly, Latino mothers of children and youth with ASD receiving special education services experience increased stress in participating in IEP meetings due to language barriers, understanding parental rights through accessible procedural safeguards, and competing family demands (e.g., lack of childcare to attend IEP meetings) [37]. Taken together with findings from this study, mothers of children and youth with ASD manage competing roles and responsibilities to coordinate the array of services their children and youth utilize and other family demands, placing them at greater risk for decreased overall health, inadequate medical home care, and lower educational service use [36].

This study is subject to limitations. Firstly, the NSCH solicits caregiver reported data, which may produce biased estimates of children and youth’s ASD diagnoses, medical home care access, and current educational service use. Secondly, the use of retrospective data (e.g., “During the past 12 months”) may be subject to recall bias. Thirdly, the cross-sectional survey design does not permit causal inferences between explanatory and outcome variables in this study. Additionally, the statistical power is limited by the small cell count of children and youth with ASD not currently receiving educational services. Lastly, this study included a relatively small ASD subpopulation despite using two age survey cohorts, which may pose limits to generalizability.

## Conclusion

We quantified the association between inadequate medical home care and current educational service use in children and youth with ASD. Findings showed inadequate medical home care was significantly associated with higher odds of current educational service use, but after adjustment, older children and youth with ASD, lower maternal health, and presenting CSHCN factors decreased the odds of current educational service use.

Our findings of higher odds of current educational service use, despite inadequate medical home care, among children and youth with ASD is promising, but warrants further study to prioritize identifiable targets within the medical home for further improving cross-sector care. Current findings suggest that interventions may be warranted for targeting medical home care provider knowledge on the relevance and scope of early intervention and/or special education services for children and youth with ASD to improve the referral component of medical home care [15], and/or by targeting family centered practices within medical home care to ease the care coordination challenges on mothers navigating different service systems for their children or youth with ASD.

## Supplementary Information

**Additional file 1: Appendix.** Multivariate Logistic Regression Models Predicting Current Educational Service Use from Inadequate Medical Home Care Children and Youth with ASD (n=1,248)

## Data Availability

The datasets analyzed for the current study were requested and downloaded from: https://www.childhealthdata.org/dataset, and administrative permission to access and use the Data Resource Center Indicator Refined 2016–2017 NSCH combined dataset was granted by Child and Adolescent Health Measurement Initiative (CAHMI). The 2016–2017 individual year datasets are publicly accessible from: https://mchb.hrsa.gov/national-survey-childrens-health-questionnaires-datasets-supporting-documents.

## Abbreviations

ASD: Autism spectrum disorder
aOR: Adjusted odds ratio
CSHCN: Children with special health care needs
CI: Confidence interval
EI: Early intervention
IDEA: Individuals with disabilities education act
IFSP: Individualized family service plan
IEP: Individualized education program
NSCH: National survey of children’s health
