# Supplementary material for: Is medical home care adequacy associated with educational service use in children and youth with autism spectrum disorder (ASD)?
Source: BMC Pediatr. 2023 Jan 9;23:12. doi: 10.1186/s12887-022-03776-3 (PMC9827663; doi:10.1186/s12887-022-03776-3)
Supplement: Supplementary file 1 — Additional file 1: Appendix. Multivariate Logistic Regression Models Predicting Current Educational Service Use from Inadequate Medical Home Care Children and Youth with ASD (n=1,248) [file 12887_2022_3776_MOESM1_ESM.docx]

**Appendix**

*Multivariate Logistic Regression Models Predicting Current Educational Service Use from Inadequate Medical Home Care Children and Youth with ASD (n=1,248)*

Odds Ratio (OR) [95% CI]

|  | **Model 1** | **Model 2** | **Model 3** | **Model 4** | **Model 5** |
| --- | --- | --- | --- | --- | --- |
| Adequacy of medical home care |  |  |  |  |  |
| Adequate | Reference | Reference | Reference | Reference | Reference |
| Inadequate | 2.19 [1.18-4.08] ^a^ | 2.15 [1.22-3.79] ^b^ | 2.10 [1.20-3.65] ^b^ | 2.10 [1.21-3.65] ^b^ | 1.97 [1.12-3.44] ^a^ |
| **Predisposing factors**  Child’s sex |  |  |  |  |  |
| Male | Reference | | Reference | Reference | Reference |
| Female | 1.55 [0.78-3.10] | | 1.53 [0.76-3.05] | 1.52 [0.76-3.07] | 1.51 [0.73-3.11] |
| Child’s age (continuous) | 0.89 [0.83-0.96] ^b^ | | 0.89 [0.83-0.96] ^b^ | 0.89 [0.83-0.96] ^b^ | 0.91 [0.84-0.98] ^a^ |
| Total children in household |  | |  |  |  |
| 1-2 | Reference | | Reference | Reference | Reference |
| ≥3 | 0.84 [0.45-1.58] | | 0.83 [0.43-1.59] | 0.82 [0.43-1.58] | 0.79 [0.41-1.52] |
| Child’s race/ethnicity |  | |  |  |  |
| White, Non-Hispanic | Reference | | Reference | Reference | Reference |
| Black, Non-Hispanic | 1.20 [0.34-4.20] | | 1.19 [0.35-4.05] | 1.17 [0.36-3.80] | 1.13 [0.34-3.76] |
| Other | 1.18 [0.55-2.50] | | 1.15 [0.54-2.43] | 1.12 [0.53-2.35] | 1.05 [0.51-2.17] |
| Overall maternal health status |  |  |  |  |  |
| Excellent or Very Good | Reference | | Reference | Reference | Reference |
| < Excellent or Very Good | 0.51 [0.28-0.92] ^a^ | | 0.50 [0.28-0.89] ^a^ | 0.51 [0.28-0.91] ^a^ | 0.52 [0.29-0.93] ^a^ |
| Adult Education Level |  |  |  |  |  |
| >High School | Reference | | Reference | Reference | Reference |
| High School | 0.60 [0.28-1.25] | | 0.56 [0.26-1.16] | 0.56 [0.26-1.22] | 0.56 [0.25-1.24] |
| Family structure |  |  |  |  |  |
| Two parents, married/unmarried | Reference | | Reference | Reference | Reference |
| Single parent, other, none reported | 1.41 [0.60-3.30] | | 1.36 [0.53-3.50] | 1.37 [0.52-3.61] | 1.29 [0.48-3.42] |
| **Enabling factors**  Insurance type |  |  |  |  |  |
| Private | Reference | | | Reference | Reference |
| Public | 1.08 [0.62-1.88] | | | 1.07 [0.61-1.87] | 0.99 [0.55-1.77] |
| Unspecified | 2.01 [0.19-21.00] | | | 1.99 [0.19-20.70] | 1.80 [0.19-16.98] |
| Federal poverty level (FPL) |  |  |  |  |  |
| >199% FPL | Reference | | | Reference | Reference |
| ≤199% FPL | 1.10 [0.59-2.06] | | | 1.10 [0.57-2.12] | 1.19 [0.60-2.36] |

a *p* < .05 b *p* < .01 c p < .001

**Appendix**

*Multivariate Logistic Regression Models Predicting Current Educational Service Use from Inadequate Medical Home Care Children and Youth with ASD (n=1,248)*

##### Enabling-vulnerability factor

Primary household languageOdds Ratio (OR) [95% CI]

##### Model 1 Model 2 Model 3 Model 4 Model 5

English Reference Reference

Spanish 1.02 [0.26-4.09] 1.11 [0.27-4.59]

Other 1.90 [0.35-10.23] 1.71 [0.32-9.20]

##### Need factors

Autism severity

Severe Reference

Mild/moderate 0.99 [0.38-2.59]

CSHCN status

ASD, CSHCN Reference

ASD, non-CHSCN 0.38 [0.17-0.85] ^a^

Age at autism diagnosis (continuous) 0.95 [0.87-1.03]

a *p* < .05 b *p* < .01 c p < .001
